# Monitoring Illegal Tree Cutting through Ultra-Low-Power Smart IoT Devices

**DOI:** 10.3390/s21227593

**Published:** 2021-11-16

**Authors:** Alessandro Andreadis, Giovanni Giambene, Riccardo Zambon

**Affiliations:** Department of Information Engineering and Mathematics, University of Siena, Via Roma 56, 53100 Siena, Italy; giovanni.giambene@unisi.it (G.G.); zambon@unisi.it (R.Z.)

**Keywords:** convolutional neural networks, internet of things, edge computing, sound classification, low power, LoRa, deforestation, illegal tree cutting, experimental tests

## Abstract

Forests play a fundamental role in preserving the environment and fighting global warming. Unfortunately, they are continuously reduced by human interventions such as deforestation, fires, etc. This paper proposes and evaluates a framework for automatically detecting illegal tree-cutting activity in forests through audio event classification. We envisage ultra-low-power tiny devices, embedding edge-computing microcontrollers and long-range wireless communication to cover vast areas in the forest. To reduce the energy footprint and resource consumption for effective and pervasive detection of illegal tree cutting, an efficient and accurate audio classification solution based on convolutional neural networks is proposed, designed specifically for resource-constrained wireless edge devices. With respect to previous works, the proposed system allows for recognizing a wider range of threats related to deforestation through a distributed and pervasive edge-computing technique. Different pre-processing techniques have been evaluated, focusing on a trade-off between classification accuracy with respect to computational resources, memory, and energy footprint. Furthermore, experimental long-range communication tests have been conducted in real environments. Data obtained from the experimental results show that the proposed solution can detect and notify tree-cutting events for efficient and cost-effective forest monitoring through smart IoT, with an accuracy of 85%.

## 1. Introduction

At the time of writing, about a third of the land on earth is still covered with forests, which play a crucial role in the planet’s environmental health. They are very important to prevent erosion and landslides, drought and to preserve the water shortage. They also purify the air, improve water quality and naturally absorb a huge quantity of carbon dioxide, thus providing a fundamental contribution to the fight against global warming and climate change [1,2].

In this context, large-scale deforestation arises together with illegal logging, thus increasing the problems of preserving global biodiversity, ecological balance, and loss of habitat for millions of wild animals. They also disrupt the global water cycle, decrease biodiversity due to habitat loss, and foster conflict and other social consequences. Despite that, each year around the world, an area greater than the whole of Italy is deprived of the forest through illegal cutting or fires, especially in developing countries such as Brazil, Indonesia, etc.

For this reason, an increase in the effectiveness of surveillance for illegal fires and logging is mandatory. On the other hand, onsite monitoring by staff patrols with on-ground control and observation towers is too expensive and time-consuming to provide capillary and pervasive monitoring due to a shortage of human resources, environmental funding, and other resources. Therefore, automatic detection techniques are needed.

In recent years, Wireless Sensor Networks (WSN) [3] have been playing a pivotal role in monitoring indoor and outdoor areas, providing interesting solutions for different scenarios [4]. Concerning WSN technologies and protocols, besides low-power short-range networks based on Zigbee and Bluetooth [5], Long Range (i.e., LoRa) [6] solutions have been introduced to achieve Low-Power Wide-Area Networks (LPWANs). LoRa offers low-power, low-cost, long-range, and long-term functionality with no maintenance, thus providing wide coverage and low battery consumption [7]. Adopting this long-range communication technology is very useful for designing and implementing capillary and pervasive sensing scenarios such as logging detection systems in forests and rural areas.

Moreover, a key point to enable the adoption of LoRa communication is the minimization of the transmitted information to reduce both the rate and energy consumption devoted to data transmission. This goal can be achieved by adopting the edge-computing paradigm, which moves the elaboration directly at the edge node, avoiding the transmission of large amounts of data in the network [8]. On the other hand, the use of computational resources (i.e., processing and memory) also introduces energy consumption, so that edge-computing has to be specifically focused on reducing the energy footprint for quite complex tasks (i.e., sensor data processing and machine learning algorithms).

Concerning sensing techniques, an interesting research topic that is being increasingly studied and addressed in the literature is event detection through audio classification. This is an innovative research field that can support environmental and wildlife preservation, ambient assisted living, and urban security scenarios. Unfortunately, the majority of systems and algorithms proposed by researchers for classifying different audio typologies (e.g., keyword spotting, human voice, environmental, urban or animal sounds, etc.) [9,10,11] are based on energy-consuming, high-computation devices such as computers, smartphones or tablets, or Raspberry Pi System on Chip (SoC) [12] devices at least. This is because it is possible to obtain excellent results in classification accuracy and robustness with high-computational resources. On the other hand, such devices are very expensive in terms of memory, computational and consequently energy resources, so they cannot be profitably adopted for pervasive or distributed monitoring (i.e., for remote areas such as a forest), where low-complexity and energy-effective smart IoT solutions are needed.

For this reason, the adoption of the edge-computing paradigm for acoustic event classification represents a further step ahead towards the extension of IoT sensory systems, by enabling IoT low-power devices to actually “perceive” a sound event like other standard environmental sensors (e.g., temperature, humidity, etc.). Thus, a trade-off between power consumption and classification accuracy needs to be carefully addressed to implement effective audio detection and classification algorithms on mobile or battery-powered low-power IoT devices. Furthermore, by retrieving GPS coordinates of the audio sensor position (e.g., by georeferencing it during set-up or by retrieving variable coordinates if moving), sound events can be spatially located to improve event detection and mapping.

In this paper, the main question of detecting deforestation due to different kinds of activities has been addressed. For this reason, we propose an innovative tree-cutting detection system based on audio event classification and wireless transmission from remote sensors disseminated in the environment to a central server, which can collect alerts (i.e., based on a specific warning-sound set, related to handsaws, chainsaws and fire sounds) and forwards them to operators for environmental protection and forest preservation.

The main contribution of the proposed study concerns the implementation of a lightweight neural network for tree-cutting audio event detection on very low-power, memory-constrained, and LoRa IoT devices explicitly designed for pervasive sound classification scenarios. The proposed solution allows for minimizing the amount of data to be transmitted over the wireless network, and consequently reduces the energy consumption of IoT devices.

This goal has been achieved by designing and testing a neural network architecture capable of performing pattern recognition by adopting pre-processed audio features on a resource-constrained ARM-Cortex M4F-powered device with only 256 kB of RAM. The proposed solution provides good accuracy and a light resource footprint on a low-cost, low-power, long-range IoT platform, thus obtaining results similar to more expensive and resource-consuming devices.

Furthermore, an experimental evaluation of LoRa coverage is provided to test the proposed system in real environments, focusing on Line-of-Sight (LoS) and Non-Line-of-Sight (NLoS) measurements in rural areas.

This paper is organized as follows: in Section 2, an overview of works related to illegal tree-cutting detection and audio classification is provided, focusing on solutions tailored for IoT devices. In Section 3, the complete solution is introduced, describing the system architecture and the main components such as audio acquisition, pre-processing, classification, and communication modules for the IoT node and the communication infrastructure. In Section 4, the implementation of a testbed is described and performance results regarding processing, classification and network coverage are reported. In Section 5, the achieved results are discussed, and finally, Section 6 reports concluding remarks.

## 2. Related Work

This section describes promising solutions found in recent literature related to audio classification techniques for the detection of tree cutting.

### 2.1. Illegal Tree-Cutting Detection

Due to the effects of illegal logging on the environment, climate change and economy, automatic detection of tree cutting has become an interesting research topic. Many studies in the bibliography aim to provide solutions to this issue. To provide sufficient low-power solutions for distributing sensors and devices in the forests, several research studies deal with motorized chainsaw detection (i.e., adopting noise thresholds), as well as vibration detection [13].

In [14], a system adopting sound and vibration sensors is presented. It uses a low-power microcontroller and an Xbee Pro S2C module for communication. The solution has been tested in small forest and open area scenarios, showing the effectiveness and efficiency of the system.

Another work based on a combination of vibration and sound sensors was proposed in [15]. The system envisaged several low-power devices and network controllers organized in fog-computing network architecture by adopting the ZigBee protocol for data transmission. By implementing suitable sleep procedures, devices can last 3 months without a recharge.

Additionally, Ref. [16] presents a device embedding both vibration and sound sensors, together with a low-power microcontroller and a GSM module to transmit the tree cutting/falling event. These sensors have been tested in several different environmental conditions to obtain suitable values for the correct behavior of the implemented threshold-based algorithm.

In [17], an innovative algorithm is presented. It detects the approximate location of chainsaws by elaborating the sound arrival-time difference related to air (i.e., through a microphone sensor) and soil (i.e., through a geophone sensor). In this way, it is possible to estimate the distance of the chainsaw from the tree/device. Furthermore, a microphone rotation can also be envisaged to obtain the actual sound direction. The proposed system can reach an accuracy of about 95% for motorized chainsaws.

The work in [7] proposed a tree-cutting system based on LoRa. As in other works, it is based on sound sensors and accelerometers, using microcontrollers and systems on a chip (i.e., Arduino and Raspberry Pi) for sensor data elaboration and GPS data acquisition. Differently from the previously described works, the adoption of LoRa technology provides several kilometers of single-hop communication range within a forest without cellular network coverage, with a battery duration ranging from 140 to 195 h.

### 2.2. Audio Classification

Besides common sensor adoption and monitoring, audio recognition could enrich automated monitoring solutions to detect events through audio classification techniques. In recent years, this innovative research topic has been gaining the interest of different researchers, and several application fields have been identified for this task, such as environmental preservation [18], wildlife monitoring [9] or urban acoustic analysis in smart city scenarios, i.e., for audio surveillance and crime detection [19]. The following key contributions from literature are described, moving from environmental sound recognition in general to solutions devoted to tree-cutting detection, with particular attention to IoT architectures.

The work in [20] provides an interesting overview of the sound classification performance of different machine-learning techniques tested on the ESC50 generic sound dataset, providing a best testing accuracy of about 72.7%. Furthermore, an example of how neural networks can perform better than manually engineered feature implementations, offering an essential contribution to audio classification, is the study described in [11], where a Convolutional Neural Network (i.e., CNN) is tested on ESC-50, ESC-10 and UrbanSound8K [21] datasets. It envisaged two convolutional layers followed by two fully connected layers, obtaining a classification accuracy performance ranging from 69% to 73% on such datasets.

Additionally, the work reported in [22] is based on CNN, implementing a three-layer architecture introducing data augmentation (i.e., Shift, Pitch Shift, Dynamic Range Compression and Background Noise) on UrbanSound8K dataset, thus obtaining an accuracy of 79%. In [23], another convolutional neural network architecture is proposed (i.e., two convolutional layers and two fully connected layers). By adopting a dilation rate in the second convolutional layer, time stretching, and noise addition, the solution achieves 81.9% accuracy on UrbanSound8K.

Such results were achieved by implementing large neural networks commonly adopted in personal computers, laptops, or other fixed computers, which are usually very expensive in terms of computational, memory and energy resources. Thus, it is impossible to exploit such systems for distributed area monitoring (i.e., in public spaces or rural area/wildlife scenarios) or pervasive health monitoring (i.e., wearable devices for ambient assisted living, animal activity tracking).

In the literature, studies on environmental sound classification also focus on resource-efficient models suitable for IoT scenarios. However, at the time of writing, most of the contributions dealing with audio recognition through edge-computing are implemented by adopting SoC hardware instead of ultra-low-power microcontrollers that are more suitable for IoT long-range and low-power (i.e., more pervasive) systems.

Specifically, the work in [24] implements cloud and fog/edge-computing for large-scale urban sound classification, testing three configurations. The first one implements both feature extraction and classification tasks within the end device (i.e., Raspberry Pi SoC). The second configuration implements feature extraction and classification on a remote server. The third configuration implements the feature extraction task in the end device and the classification task on the server. Tests were conducted on classifier performance, power consumption and runtime, and the hybrid (i.e., third) configuration obtains the best overall results.

The work in [25] adopts several 1D CNN of three to five layers to reduce computation and memory requirements, accepting directly audio samples in inputs instead of 2D time–frequency representations (i.e., spectrograms). This solution envisages fewer parameters than a typical 2D CNN already present in the literature [11,22], thus achieving 89% accuracy on Urbansound8K using Gammatone filters. Unfortunately, this solution still requires 550,000 parameters, which is not feasible with IoT ultra-low-power and memory-constrained microcontrollers such as ARM Cortex M4/M4F boards.

The work described in [26] implements audio event detection by optimizing deep-learning techniques, mainly focusing on knowledge distillation and 8-bit quantization, tailored to resource-constrained low-power devices. It also demonstrates that an embedded neural network framework such as CMSIS-NN [27] can be useful to speed up processing. The proposed model size is only 34.3 kB so that it can be implemented on an ARM Cortex M4 processor. However, it achieves a classification accuracy of about 68% on Urbansound8k.

Concerning the illegal tree-cutting detection in forests, besides vibration and noise threshold sensors, several audio detection frameworks have been proposed to face this issue. The work in [28] is based on the extraction of Haar-like features from a spectrogram to detect chainsaw sounds, working on the frequency domain. A two-stage threshold-based approach is adopted to discriminate between chainsaw and non-chainsaw sounds. Results show that the solution can effectively recognize chainsaw sound if the sound signal is stationary over time; this is not the case in general. In that work, no information has been provided on adopted computation hardware and communication modules.

In [29], three algorithms were tested to detect the tree-cutting event: Gaussian mixture model, K-means Clustering, and Principal Component Analysis. Furthermore, the work proposes a new algorithm achieving the best accuracy, reaching 92%. Unfortunately, the paper does not provide any information on the adopted data communication protocol if implemented.

In [18], several monitoring stations (equipped with microphones) are disseminated in the forest. They record sounds and forward (through Wi-Fi or ZigBee wireless communications) the acquired samples to a server for audio processing and classification of the incoming sound. At the server, detection has been performed through the adoption of neural networks envisaging pretrained logging models that obtain a recognition accuracy of about 94.4%. However, the adoption of server-side classification and short-range wireless protocols cause an expensive implementation for a large-scale system.

In [30], the proposed system detects and locates chainsaws in the forest. It envisages a three-tier architecture based on the Time Difference Of Arrival (TDOA) and multilateralism. To correctly identify the chainsaw audio event, a neural network trained with a self-collected chainsaw dataset has been implemented on the adopted Raspberry Pi SoC, obtaining an accuracy of about 96% on a small testing dataset. For communications, the 802.15.4 protocol has been adopted.

Table 1 reports a schematic overview of the works focused on automatic tree-cutting recognition. This table highlights the limitations of each contribution with respect to the solution proposed in this paper. Audio classification systems focused on more general scenarios (i.e., urban sounds) have not been included.

## 3. System Description

The system proposed in this paper combines the long-range coverage of LPWAN protocols with an efficient CNN-based audio classification system for automatic detection of tree cutting on IoT low-power edge-computing nodes. It is composed of an application server that is connected to several LoRa gateways, and each gateway covers a wide area where end nodes are disseminated.

**Figure 1:**
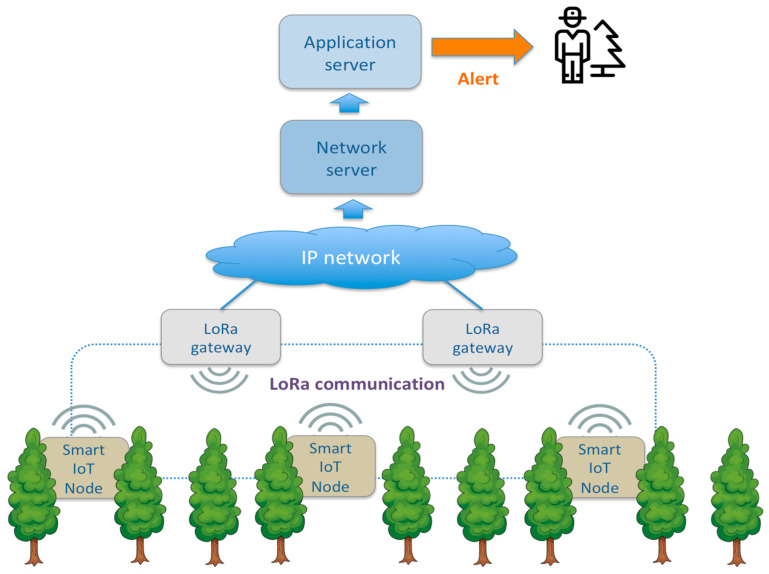
System architecture.

Each end node monitors the environmental audio to distinguish between common sounds (e.g., rain, birds, wind) and sounds related to illegal cutting of logs or other dangers (e.g., a fire) to classify them correctly. In case of detecting sounds related to fire or tree-cutting behaviors, the node sends a message to the server by transmitting a LoRa packet data received by the LoRa gateways in range. After packet reception, the gateways forward the data received from the end nodes in range to the LoRaWAN network server through an IP network (i.e., adopting a fixed backhaul or an LTE connection). Finally, the application retrieves the information from the network server in a LoRaWAN paradigm. Figure 1 provides an overview of the proposed architecture.

The real-life operating scenario for the proposed system envisages the dissemination of several IoT nodes in the monitored forest under the wireless coverage of one or more gateways in a star-of-stars topology typical for LoRa technology. Nodes and gateways can be placed on the trees or a dedicated camouflaged pole for forest-sound listening. Suppose someone nearby is using a handsaw or a chainsaw for tree-cutting activity, or fires up a blaze, in that case, the device detects such specific sound by notifying it to the application server, which alerts whoever is in charge of forest conservation.

Concerning solutions where the IoT node only captures and retransmits the audio data, this IoT architecture has the advantage that feature extraction and audio classification through a neural network are performed directly on the IoT node according to an edge-computing paradigm. In this configuration, only the event notification has to be transmitted with an extremely low bandwidth occupation (i.e., 99.99% transmission stand by). This bandwidth efficiency enables the adoption of a LPWAN communication protocol such as LoRa, which provides long-range coverage and very low-power consumption for low-data-rate transmissions.

On the other hand, concerning other edge-computing solutions for audio classification provided in the literature, the proposed system provides audio classification capabilities on resource-constrained hardware (i.e., with a low-power ARM Cortex M4F microcontroller), which is less complex, smaller and has very low electric power demands for common hardware devoted to machine-learning tasks, thus enabling low maintenance and sustainable power supply through renewable solar sources, i.e., for remote location scenarios.

**Figure 2:**
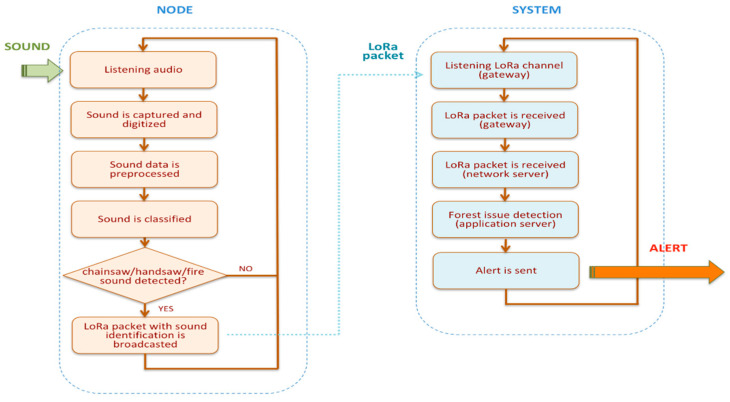
Operational flowchart.

A flowchart depicting the main operations performed by the smart IoT node and the system is shown in Figure 2.

### 3.1. IoT Node Design

**Figure 3:**
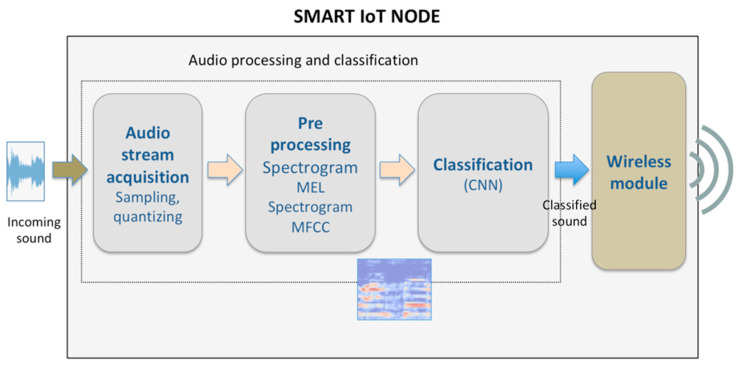
System design—LoRa end node.

The monitoring system implemented in the LoRa end node is composed of four key elements that are depicted in Figure 3:

- an acquisition module, which performs sampling and quantization of the incoming sound;a pre-processing module for data representation and feature extraction;
- a classifier, based on CNN;
- a long-range low-power wireless communication module for delivering the notification of the audio classification response to a remote device (such as a gateway).

The first three elements described above compose the audio processing and event classification subsystem.

### 3.2. Wireless Communication

To obtain a wide coverage, the long-range low-power LoRa wireless transmission protocol is adopted for data communication between end nodes and gateways. LoRa is a physical layer technology operating in Industrial, Scientific and Medical (ISM) radio bands at 433 MHz, 868 MHz, and 915 MHz frequencies [6]. Furthermore, the LoRaWAN open protocol is implemented for the MAC layer, providing a data rate between 0.25 to 5.5 kbps depending on bandwidth, spreading factor, and coding rate, with wide coverage and low battery consumption [7]. The LoRaWAN standard envisages a network architecture organized in a star topology, foreseeing gateways that receive messages from end devices (i.e., IoT edge nodes) through LoRa. Then, gateways convey the data to a network server by adopting an IP-based fixed or wireless (i.e., fiber, satellite, 4G, 5G) backbone connection [31].

In LoRa, an end device adopts an ALOHA-like protocol to access the radio channel. This behavior reduces complexity but introduces collisions if many packets are sent at the same time from several devices [32]. By adopting LoRaWAN, multiple copies of packets (which can be emitted by one node and received by several gateways in range) are filtered before forwarding data to the application server. Finally, the envisaged network server provides data security and privacy.

Due to its low-power and long-range characteristics, the LoRa technology has been adopted in many different application scenarios envisaging IoT, such as smart parking systems [33], fall-detection scenarios [34], health monitoring in rural areas [35], forest preservation [36], object tracking [37], smart city and smart farming applications [38,39]. On the other hand, other long-range technologies such as NarrowBand IoT (NB-IoT) can also be easily integrated into the IoT device if LTE coverage is available in the monitored area [40].

### 3.3. Incoming Sound Acquisition

Concerning the audio classification process, the first block depicted in Figure 3 deals with sound acquisition and digitalization, which are carried out through an embedded microphone and suitable digital audio acquisition hardware, respectively. The detected audio signal is sampled at a specific sampling frequency (i.e., 16 kHz or 44.1 kHz) and then is quantized at a certain bit depth (i.e., 8, 16 or 32 bits).

Since compression artifacts can create some problems during the machine-learning classification process, a WAV PCM [41] format can be adopted with FLAC [42] lossless compression audio codec.

As the sampling frequency (i.e., related to the total number of samples describing the acquired sound) and the quantization depth have a direct impact on the dimensionality of the input and therefore on memory and computational cost of the model, the system proposed here acquires a single audio channel of the incoming sound and of the clips stored in the dataset, using a sampling frequency of 16 kHz. As shown in the testing section, this choice allows for reducing resources in terms of memory, computation and energy, without affecting system accuracy. Therefore, it represents a good trade-off between the good quality of the input and a sustainable computational cost of the classification model, especially for environmental audio sampling [25]. Furthermore, to test the trade-off with respect to quantization, the audio samples have been quantized at two different bit-depth values: a common depth of 32 bits per sample and a more compact representation of 8 bits per sample.

**Figure 4:**
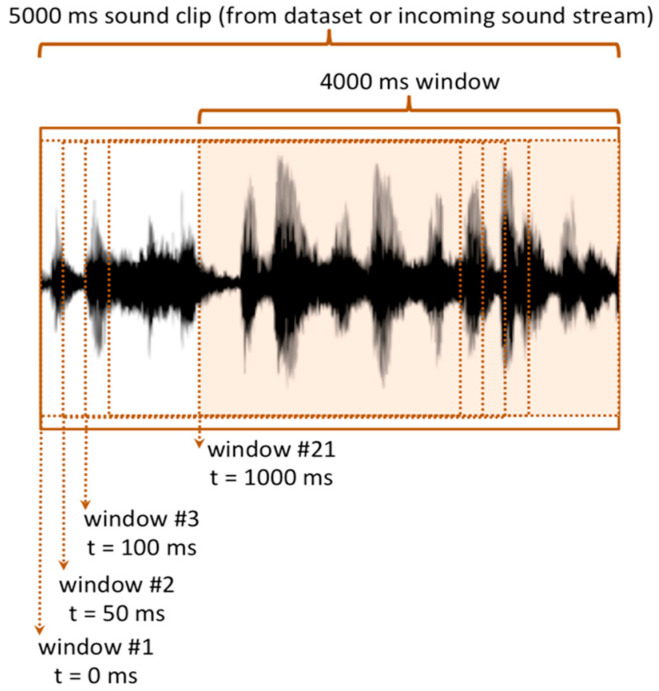
Sound acquisition and temporal sliding windows.

Feature extraction related to the obtained samples is carried out by collecting them in temporal window frames of 4000 ms; this duration appears to be suitable for representing most of the sounds of the dataset. Moreover, during the acquisition process, the incoming sound is divided into fixed-length frames so that the samples are sliced up into several overlapping temporal windows, as shown in Figure 4, where each window is shifted by a certain offset (i.e., 50 ms) from the subsequent one. According to a sliding window technique, an audio clip lasting 5 s results in 21 distinct overlapping windows of 4 s, each one shifted by 50 ms. This overlapping process naturally introduces a number of reused audio signal samples, thus providing a basic data augmentation, which is helpful for improving the training accuracy [25].

### 3.4. Pre-Processing

Environmental acoustic sounds envisaged in this work, and other acoustic sounds in general, are composed of nonperiodic signals. In this case, the frequency representation obtained through Fast Fourier Transform (i.e., FFT) is not sufficient, and a specific representation of the sound in both time (temporal signature) and frequency (spectral signature) domains has to be provided. For this reason, these sounds need to be analyzed before the classification task in a time–frequency representation by computing a sound spectrogram from the incoming audio samples, thus obtaining an image (i.e., matrix) describing the contribution of various frequencies in an audio signal across time. In this work, three pre-processing techniques have been considered to better represent the incoming audio signal and highlight its features for more accurate classification. In the following, a brief description of the three methods is provided.

#### 3.4.1. Linear Spectrogram

A spectrogram is a time–frequency visual representation of the spectrum of frequencies composing a signal as it varies with time. A linear spectrogram is generated through Short-Time Fourier Transform (STFT) [43], splitting the audio into consecutive short slices and then calculating the FFT on each slice. The absolute value of the magnitude of the obtained complex value is squared and the phase information is discarded.

Concerning the implementation described in this paper, the audio window frame has been split into subframes of 20 ms, with a subframe stride of 10 ms. The linear spectrogram has been computed adopting 256 frequency bands.

#### 3.4.2. Mel-Scaled Spectrogram

**Figure 5:**
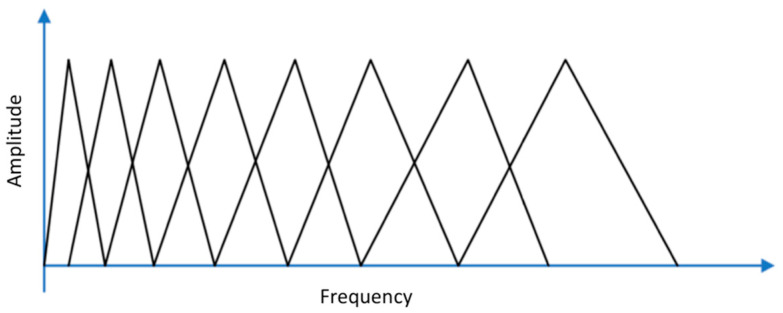
Mel scaled filterbank.

To save resources for training and reduce inferencing times, it is important to diminish as much as possible the dimension of the input of the machine learning module. Adopting a Mel-scaled filterbank [44], consisting of a sequence of triangular audio signal filters, allows for cutting down the correlation between adjacent frequency bins of the linear spectrogram (the correlation is related to information redundancy). A Mel spectrogram is the result of applying a Mel-scaled filterbank (see Figure 5), and its representation behaves correctly for general audios [45,46].

As for the linear spectrogram, the audio window frame has been processed adopting subframes of 20 ms of duration, subframe stride of 10 ms and 256 FFT values. Furthermore, to reduce the correlation, a Mel-scaled filterbank composed of 32 filters has been adopted.

#### 3.4.3. Mel Frequency Cepstral Coefficients

Mel Frequency Cepstral Coefficients (MFCC) is a way to represent an audio signal in a compact form, and it can be useful for edge-computing classification on IoT devices with limited resources. It allows for extracting sound features from Mel spectrograms efficiently. Mel Frequency Cepstral Coefficients are generated by applying a Discrete Cosine Transform (DCT) to a Mel spectrogram to reduce the input size for the neural network classification phase.

With this technique, it is possible to obtain a good trade-off between performance and computational and memory cost for audio classification [44,47,48], reducing dimensionality to only 13–20 coefficients with low correlation.

Using this pre-processing technique, the audio window frame has been processed adopting subframes of 20 ms, subframe stride of 20 ms, 256 FFT values and a Mel-scaled filterbank with 32 filters. Finally, 13 cepstral coefficients have been used for data representation.

### 3.5. Classification

The classification process envisages two phases: a training phase, where a model learns through the provided training dataset the characteristics of sound classes by training the neurons of the CNN; and an inference phase, where a new audio data (i.e., live-acquired and pre-processed sound) is classified within N predetermined (i.e., learned) classes basing on its specific characteristics.

To obtain a lightweight and efficient neural network, in this work, the sound classification task is performed by implementing a CNN, an architecture often adopted in literature due to its good performance on audio classification [22,49].

**Figure 6:**
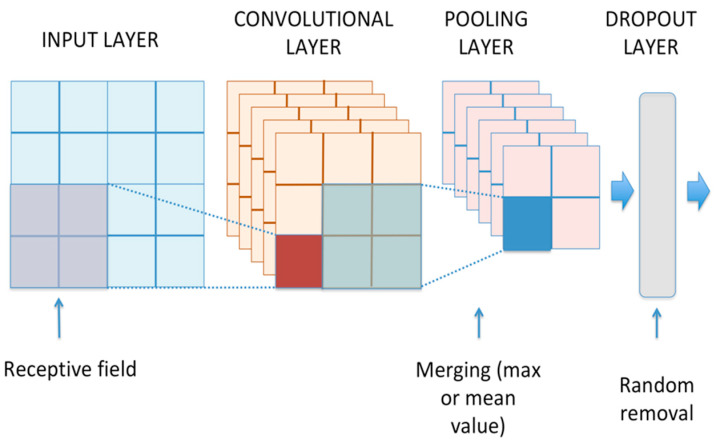
Convolutional neural network layers.

In a convolutional neural network, the audio features representing the input data have been processed through several trainable convolutional layers combined with pooling and dropout layers (see Figure 6) to obtain an appropriate representation of the input [11]. In contrast to Multi-Layer Perceptron Neural Networks (MLPNN), a convolutional layer takes advantage of the local structure present in the input data. Specifically, neurons belonging to a specific layer are connected only to a small region (i.e., receptive field) of the previous layer, according to the local connectivity theorem [50]. This approach allows for reducing the estimation parameters and improves the invariance to translational shifts of the data.

Further invariance and dimensionality reduction can be achieved by merging the outputs of layer neurons into a single neuron in the next layer by calculating the max or the mean value. This reduction is achieved by introducing pooling layers between convolutional layers to increase the area covered by receptive fields. Dropout layers are envisaged to randomly (i.e., adopting a predefined probability) remove some hidden units, thus preventing overfitting (i.e., the loss of the underlying structure of the input data) as much as possible during the training phase [51]. Lastly, the final convolutional layer output is then flattened to be ready for the classification process.

**Figure 7:**
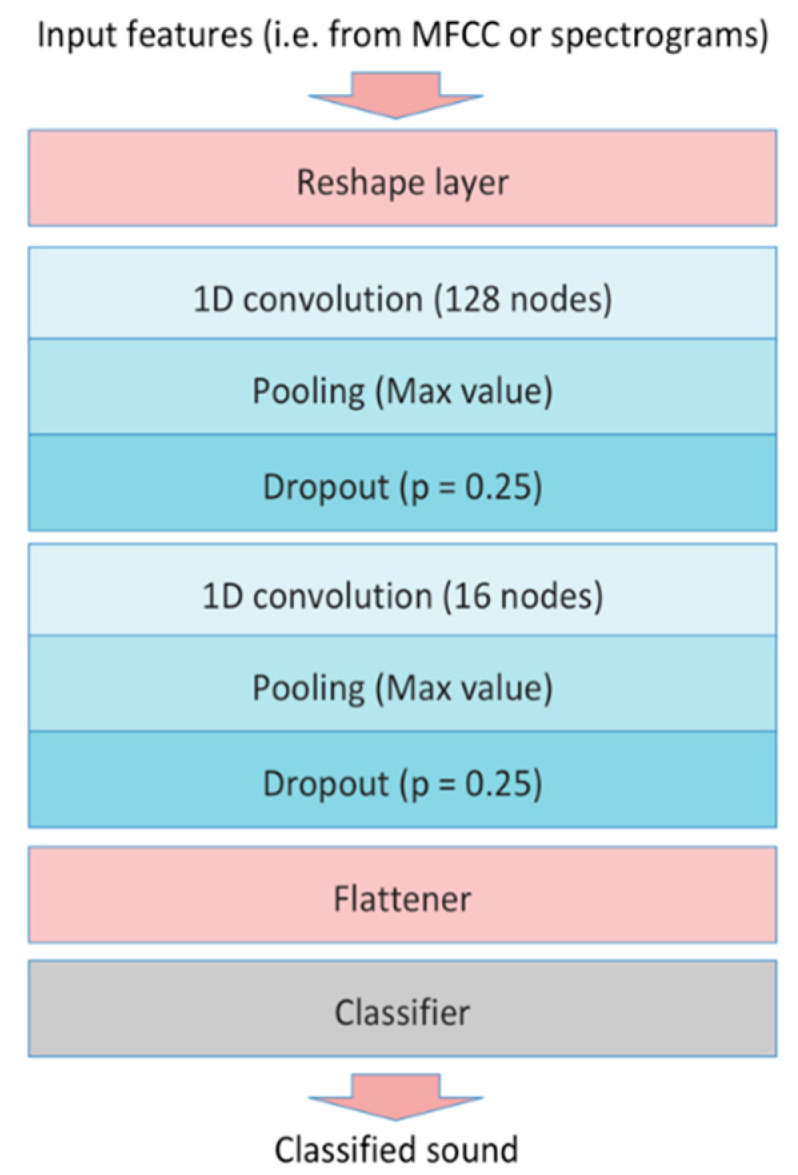
Neural network configuration.

This work adopts a configuration based on a convolutional neural network (Figure 7). As a first block, it envisages a shaping layer to manage the input features. Then, a 128-neuron, mono-dimensional convolutional layer is implemented. To reduce dimensionality, after this layer, a pooling layer (i.e., calculating the max value) is added. Moreover, to reduce the risk of model overfitting during the training phase, a dropout layer (i.e., adopting a dropout probability of 0.25) is envisaged. Furthermore, a second block of three layers has been implemented (i.e., a mono-dimensional, convolutional, 16-neuron layer, a max-pooling and a dropout layer envisaging the same drop probability). Finally, a flattening layer manages data before classification. The neural network configuration is depicted in Figure 7.

### 3.6. Sound Dataset

The dataset adopted for the tree-cutting detection is a subset of the ESC50 dataset [20], obtained by selecting specific sound classes related to the forest environment to obtain a more realistic dataset (i.e., wind, chirping birds and crickets, rain). Concerning sound classes related to dangerous situations for forest preservation, besides the classical chainsaw sound, we have also trained the system to recognize the sound of handsaws and fires. Specifically, the adopted dataset comprises the following seven sound classes: chainsaw, chirping birds, crackling fire, crickets, handsaw, rain, wind.

For each sound class of the ESC dataset, 40 recorded clips (5-s-long duration) are foreseen, for a total registration time of 23 min and 20 s (i.e., 17 min and 30 s for training, 5 min and 50 s for testing). To separate clips for training and testing sessions, 10 of the labeled clips in a class have been randomly chosen as testing data and the remaining 30 as the training set for each sound class in the dataset.

To reduce RAM and processing time needed for spectrograms and MFCC computation, the adopted hardware and its sound acquisition system use a sampling frequency of 16 kHz. Therefore, the clips were downsampled from the original 44.1 kHz to 16 kHz to be correctly compared to the live sounds recorded by the embedded microphone.

### 3.7. Trade-Off between Resource Consumption and Classification Accuracy

Although CNN can already be considered a lighter neural network with respect to MLPNN, further modifications have to be implemented to reduce memory and processing resource consumption. As a first step, network quantization [52] can be performed by using 8-bit integers instead of 32-bit floating-point. In image classification tasks shown in the literature, quantization provides improvements of about 4.6× in runtime and 4.9× in energy savings [27]. Moreover, in the work described in [53], researchers show that the loss of precision related to quantization cannot considerably affect the final accuracy.

Furthermore, several neural networks are implemented by adopting a generic interpreter (i.e., as for TensorFlow Lite for Microcontrollers embedded runtime). However, the compilation of the neural network directly in C++ source code avoids the need for an interpreter, thus reducing code instructions. This efficiency enhancement can be obtained by adopting an embedded-C neural network framework, the CMSIS-NN library, which implements mono-dimensional and bi-dimensional convolution layers and pooling layers by adopting fixed-point or integer variables [27,54]. This library is specifically designed to increase the performance of learning kernels on Cortex m processors to provide a basic and energy-efficient version of CNNs.

In this work, the CMSIS-NN library has been adopted; the efficient quantization allows the adoption of a low-power ARM Cortex M4 processor with a good performance, as shown in Section 4.

### 3.8. Prototype

**Figure 8:**
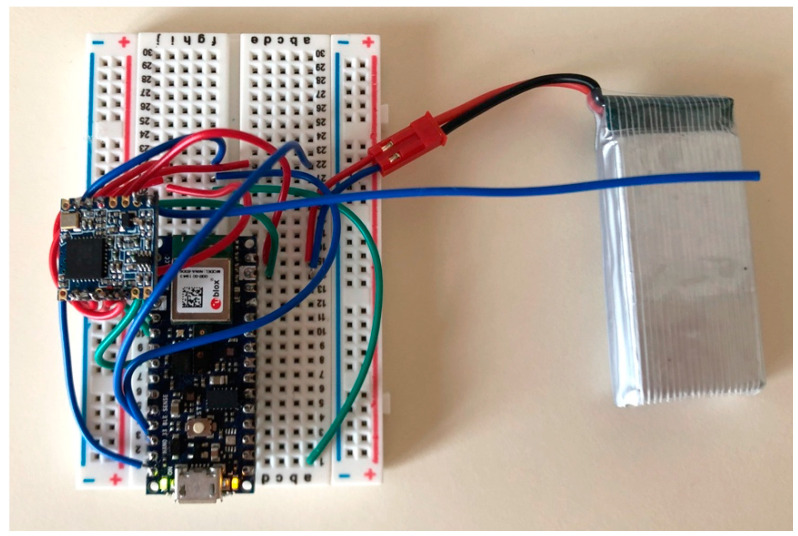
Prototype of the monitoring device.

The prototype of the proposed IoT sound classification node is shown in Figure 8. The hardware consists of a 32-bit ARM Cortex MF4 chipset running at 64 MHz, with memory storage of 1 MB on Flash and 256 kB of SRAM.

The ARM Cortex MF4 processor can implement machine-learning features with very low energy. A Li-Po battery pack of 3.7 V-1800 mAh powers the device. The system also embeds an omnidirectional microphone for environmental audio acquisition.

As for wireless communications, the device implements a LoRa technology module, which broadcasts the information related to the inferred sound to gateways in range to reach the network and the application server. The LoRa communication is set at the maximum power level allowed in Europe, with an uplink power of 25 mW (i.e., 14 dBm) and frequency of 868.1 MHz, spreading factor 7, a bandwidth of 125 kHz, coding rate = 4/5, payload = 8 bytes, and preamble length = 8 bytes. A transmission delay of the LoRa packet at each end node has been envisaged to avoid on-air data collision. This delay has been quantified in number of milliseconds proportional to the matching percentage probability of the incoming sound with a chainsaw, handsaw, or fire class for the neural network on that node (as an example, a matching probability of 87% results in a transmission delay of about 870 ms), but any other suitable randomization technique can be adopted.

## 4. Results

This section reports the results related to pre-processing performance and classification accuracy. It describes the experimental testbed implemented in a rural area with woods and several buildings to evaluate LoRa wireless network coverage and energy consumption of the IoT device.

### 4.1. Pre-Processing

First, the samples have been correctly acquired through sampling and quantization processes by adopting the sliding window technique, and then they have been elaborated by the pre-processing phase to extract features for more efficient classification. Specifically, for each sample window, pre-processing operations have been performed according to linear spectrogram, Mel spectrogram, or MFCC techniques.

Table 2 reports a performance comparison between different pre-processing techniques in terms of processing time and peak RAM used to process the incoming sample window with the adopted hardware.

The table shows that the processing required for linear spectrogram computation needs about half of the time with respect to Mel-spectrogram computation. On the other hand, the peak RAM usage for the first pre-processing technique is double that of the second one. Concerning the MFCC pre-processing technique, it entails a very low memory usage for computation with respect to both spectrogram methods, requiring only 46 kB of RAM with a processing time comparable with the fastest technique (i.e., 928 vs. 714 ms). For this reason, MFCC pre-processing represents the best trade-off between low RAM usage and good processing time, the overall classification time requirement being not so stringent.

### 4.2. Neural Network

A performance comparison among different pre-processing methods is presented in Table 3 in terms of inferencing time, peak RAM usage, ROM usage and accuracy (referring to the adopted 64 MHz ARM Cortex M4F processor). Results have been obtained on the testing set by implementing CNN with pre-processed inputs with Linear Spectrogram, Mel-Spectrogram and MFCC techniques, respectively, and by adopting a quantization bit depth for the audio acquisition of 32 bit (i.e., floating-point) and 8 bit (i.e., integer), respectively.

Table 3 highlights that the MFCC solution outperforms all the others, and a significant reduction in memory resources and response time (i.e., 4.5× inferencing time, 3.8× peak RAM, 2× ROM) can be achieved through the adoption of 8-bit-depth integers instead of 32-bit-depth floating-point variables, with a negligible loss of accuracy.

**Figure 9:**
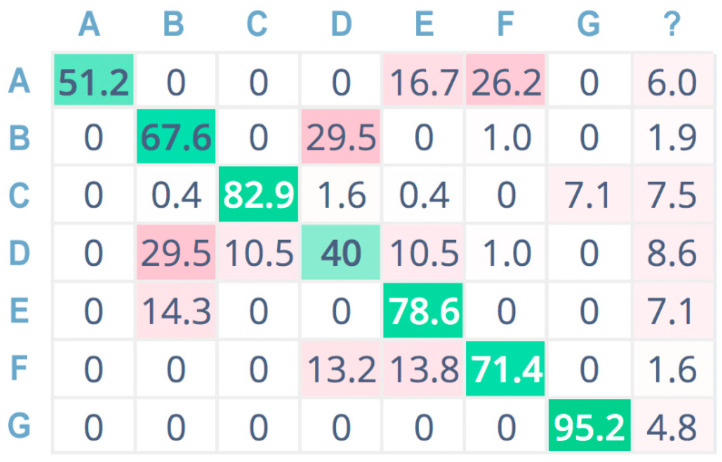
Confusion matrix adopting linear spectrogram pre-processing, 32 bit.

**Figure 10:**
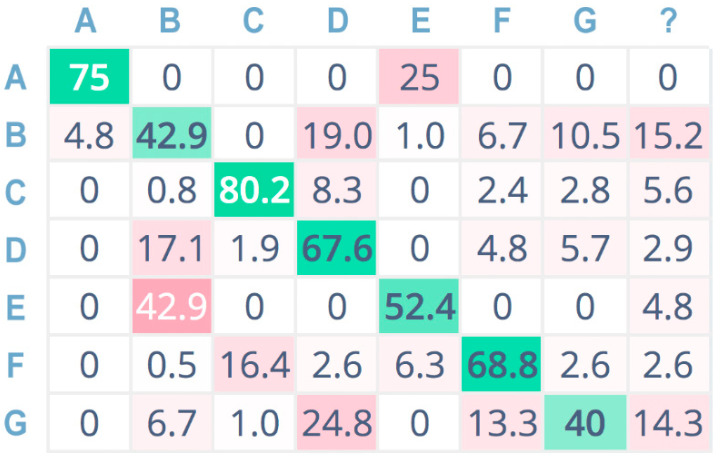
Confusion matrix adopting Mel spectrogram pre-processing, 32 bit.

**Figure 11:**
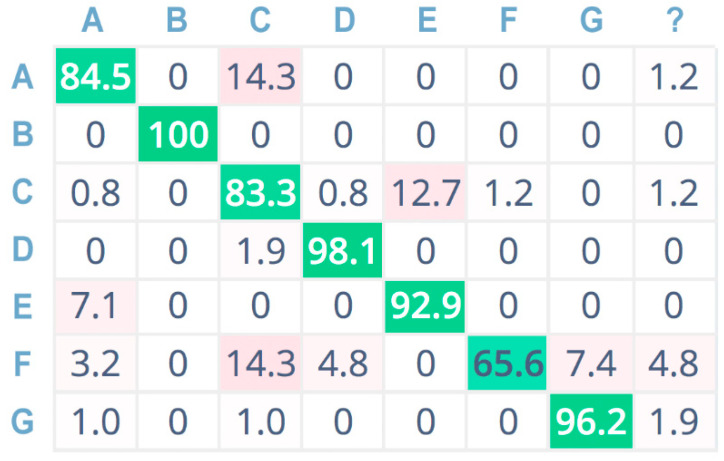
Confusion matrix adopting MFCC pre-processing, 32 bit.

Furthermore, Figure 9, Figure 10 and Figure 11 show the confusion matrices related to the tests performed on the seven sound classes of the dataset, adopting 32-bit-depth quantization with a linear spectrogram, Mel-spectrogram, and MFCC pre-processing techniques, respectively. A confusion matrix is composed of cells, where the cell’s row index *i* indicates the original class and the column index *j* reports the class inferred by the classifier. Each cell value reports the percentage of times for which class *i* has been inferred as *j*. The diagonal cells (depicted in green color) represent the correctly classified instances, and consequently, they provide an indication of the accuracy value; on the contrary, the other cells (in red color) represent misclassified instances [55]. Color (i.e., green or red) intensity grows with the classification instance counts (i.e., correct or incorrect) for the specific cell. The last column is related to undetermined instances (i.e., uncertain classification, labeled with a question mark).

For a more compact representation, sound classes are indexed as follows: A = chainsaw, B = chirping birds, C = crackling fire, D = crickets, E = handsaw, F = rain, G = wind.

The confusion matrix related to CNN classification adopting a linear spectrogram (Figure 9) shows good accuracy only for a few classes: the wind (G), crackling fire (C), and handsaw (E) classes.

Instead, the worst-accuracy performance falls on the crickets (D) class, misclassified as chirping birds (B).

The confusion matrix related to CNN envisaging Mel spectrograms (Figure 10) shows less overall accuracy, with acceptable values only for the chainsaw (A) and crackling fire (C) sound classes. However, A and C are two of the key sound classes for tree-cutting detection. On the other hand, the third class related to forest preservation issues is the handsaw (E) sound class, which has a high misclassification probability, especially regarding misclassification as the chirping birds (B) sound class.

The neural network achieves the highest overall testing accuracy by adopting MFCC as a pre-processing block (Figure 11). Specifically, it performs very well for the chirping birds (B) class and the crickets (C) class. The rain (F) class has the lowest accuracy value, but considering that the values indicated in the other columns of the F row are much lower, this class has a low misclassification probability. However, concerning the chainsaw (A), crackling fire (C), and handsaw (E) sound classes, it obtains high accuracy values (i.e., 84.5%, 83.3%, and 92.9%, respectively), offering a reliable solution for log-cutting detection and forest preservation.

**Figure 12:**
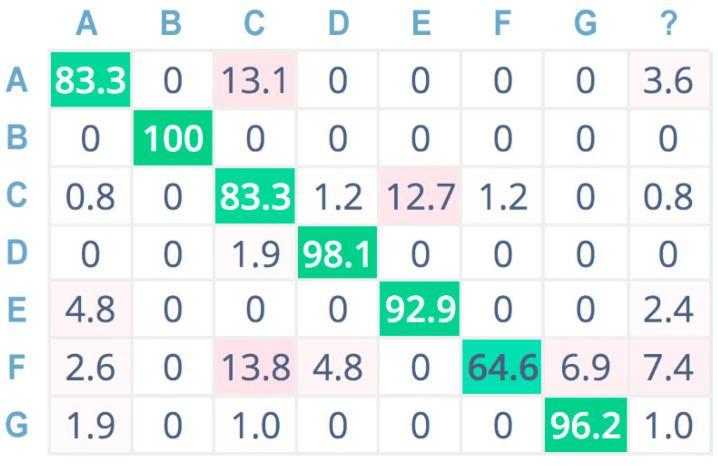
Confusion matrix adopting MFCC pre-processing, 8 bit.

Figure 12 also reports the confusion matrix related to 8-bit-depth quantization for the MFCC pre-processing technique. It is easy to see that the accuracy is almost the same obtained with 32-bit-depth quantization for MFCC. Only for chainsaw (A) and rain (F) sound classes, the 8-bit solution shows a slight decrease in accuracy (i.e., −1.2%). However, the CNN implementation adopting MFCC as pre-processing technique and 8-bit audio signal quantization depth provides an average testing accuracy which is still greater than 85%, while dramatically reducing computational, memory, and power resource consumption with respect to the same 32-bit pre-processing technique and other solutions with lower accuracy values such as the 8/32-bit linear spectrogram and Mel spectrogram.

### 4.3. LPWAN Communication

The LoRa wireless communication performance test has been carried out according to LoS and NLoS scenarios and it has been focused on studying the LoRa coverage by measuring the RSSI at the gateway.

#### 4.3.1. Testing Scenario Description

**Figure 13:**
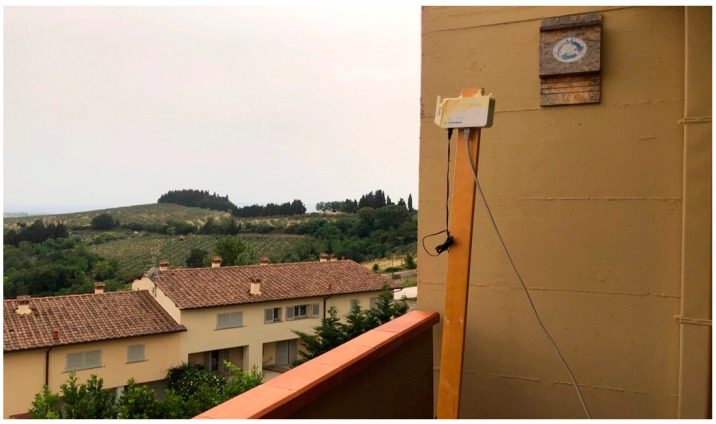
Gateway setup.

**Figure 14:**
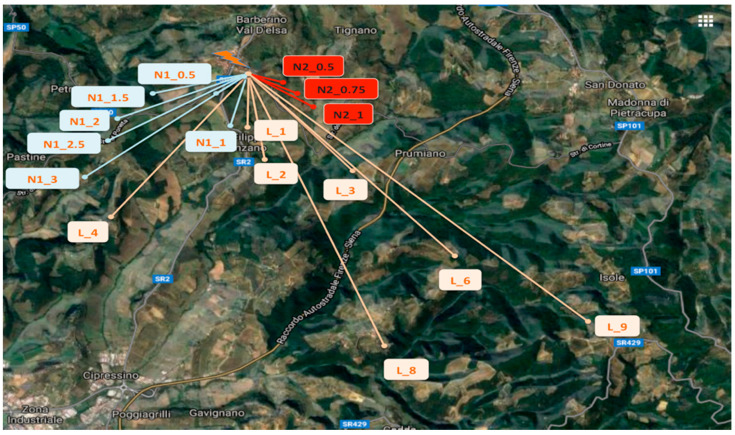
LoS and NLoS tests, map.

**Figure 15:**
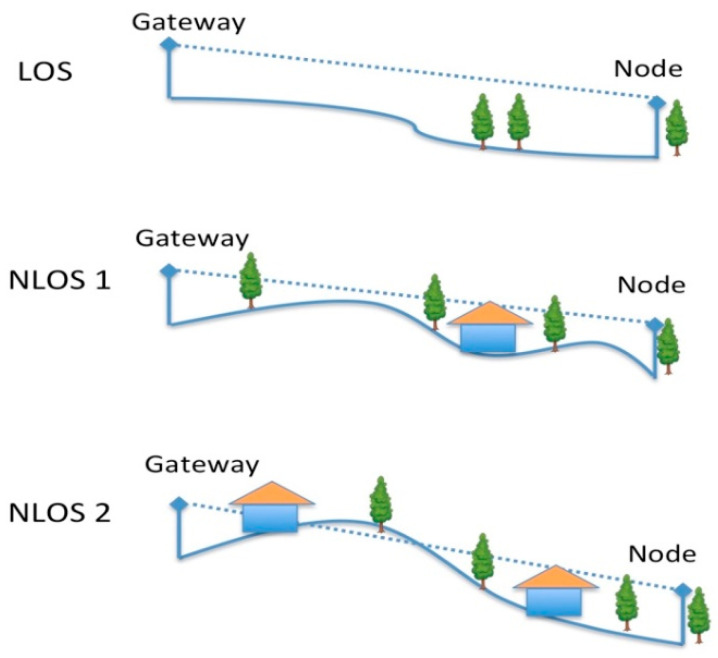
LoS, NLoS1 and NLoS2 cases.

The testbed was implemented in a rural area of Chianti, in Italy. The testing area is located on hills where there are olive groves, vines, some woods, and small and sparse buildings. The LoRa gateway was positioned on a terrace of a building located on a south slope of a hill (at 2 m height from the terrace floor and 6 m from the lower surface of the building), as depicted in Figure 13. The testing node, transmitting at 14 dBm, was placed in the environment (see Figure 14) at different positions to cover the following cases (see Figure 15):

- LoS case: no obstacles were placed between the gateway and the node with sensors;
- NLoS1 case (partial blockage), where some obstacles (i.e., woods, rural buildings, groves, small hills) between the node and the gateway can cause shadowing and scattering;
- NLoS2 case (full blockage), where huge buildings and hills are present between the gateway and the node.

#### 4.3.2. Results

Table 4, Table 5 and Table 6 show the RSSI values obtained for the different node locations as depicted in Figure 14, grouped for LoS, NLoS1, and NLoS2 cases, respectively. The values shown are average values calculated on 10 repeated measurements obtained for each location. Tests were conducted under good weather conditions (i.e., sunny weather).

In the LoS case, the coverage radius reaches more than 8 km, which is in line with the results obtained in [56]. Furthermore, the RSSI values obtained for the different distances are similar to the ones obtained in [56] for the Spreading Factor 7 case, which differs from the Okumura-Hata model of about 27 dBm. The coverage range is reduced at 2.5 km in the case of shadowing and scattering effects of the NLoS1 case. The coverage radius is lower than 1 km if the node is located on the other side of hills, as in NLoS2 cases. Finally, being the proposed solution focused on forest preservation, a LoRa coverage test was also performed by placing the end node inside a small forest to calculate the RSSI values considering the attenuation of real oak woods as a starting point for sensor positioning in larger forest areas.

**Figure 16:**
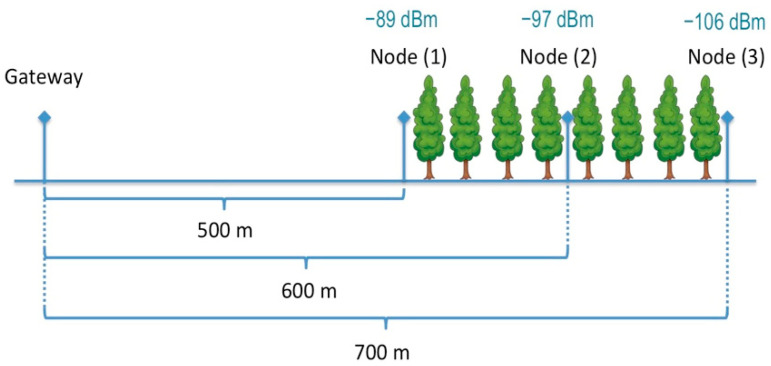
Forest case.

The scenario is briefly depicted in Figure 16, also showing the RSSI values obtained at the beginning of the wood area (i.e., −89 dBm, location 1), in the middle (i.e., −97 dBm, location 2), and on the other side of the wood (i.e., −106 dBm, location 3), thus envisaging an average path loss related to woods of about −8.5 dBm/100m. Additionally, in this case, values have been averaged on repeated measurements. Experimental values show an attenuation rate similar to the one obtained in the ITU report related to attenuation in vegetation [57] for radio communications below 1 GHz in woodland (i.e., between −8 and −10 dBm every 100 m).

### 4.4. Energy Consumption

The end-node energy consumption for continuous sound monitoring and classification (with consequent communication through LoRa if the inferred sound overcomes the 0.8 probability) by envisaging MFCC pre-processing and 8-bit integer variables has been measured. Tests have been conducted using an 1800 mAh 3.7 V battery.

By considering the tree-cutting notification as a rare event, the proposed device has been able to perform sound acquisition, classification, and communication (i.e., providing alerting through LoRa) for more than 61 h continuously (with an average current intensity of about 29.5 mA) without battery replacement or recharge.

## 5. Discussion

The results described in Section 4.1 and Section 4.2 show that sound classification on a tightly resource-constrained (i.e., low-power) device is not a trivial task. The design of neural networks devoted to this purpose must take into account the shortage of RAM and ROM resources and the low computational power that can affect the overall classification time. Specifically, it is evident that pre-processing methods based on a linear spectrogram and a Mel spectrogram envisage more than double and quadruple peak RAM usage when compared with MFCC.

A tiny CNN has been explicitly designed to achieve a good trade-off between accuracy and computational/memory resource use. Furthermore, by adopting this neural network, the choice of spectrogram pre-processing requires high computational and memory resources to obtain sufficient accuracy values (i.e., 71.77%, by adopting 32-bit quantization); therefore it is not sustainable for the hardware architecture envisaged for low-power IoT monitoring.

On the other hand, solutions based on MFCC pre-processing need lower resources with higher accuracy values, thus significantly reducing the misclassification probability. Although the MFCC solution adopting 32-bit quantization reaches the highest accuracy value, it is possible to obtain the highest efficiency with the 8-bit integer quantization. As already shown in Table 3, the last solution provides a very low RAM/ROM usage and inferencing time, becoming a state-of-the-art solution for sound classification on tightly resource-constrained devices.

Concerning wireless communications, tests performed in Section 4.3 show that LoRa is a feasible communication technology that can provide long-range communication of more than 8 km in LoS scenarios and more than 2.5 km in a light NLoS scenario. However, as shown by tests carried out inside a wood, the communication distance is reduced due to the density and thickness of the woods located between the transmitter and receiver. In the tested configuration, attenuation provided by oak forest is calculated at about 17 dBm/100 m, envisaging an estimated coverage radius lower than 1 km. Similar experiments adopting other LPWAN technologies, such as NB-IoT will be investigated in future works.

Moreover, by transmitting alert information only when a sound associated with a dangerous event (i.e., chainsaw or fire or handsaw) is detected, the designed low-power edge-computing device allows for over 61 h of continuous sound classification activity without the need for battery recharging, paving the way for photovoltaic-based charging for low-maintenance IoT devices with LoRa transceivers.

As for the system response times, they are composed of the processing time and transmission time. An overall processing time (i.e., from incoming sound digitalization to final classification) of 1160 ms is obtained with the most promising solution adopting MFCC pre-processing and 8-bit quantization. Lower detection times would be possible by using more powerful hardware for the sensors. However, this solution would inevitably lead to greater power consumption that would not adapt to our context, where sensors with the related processing must work in remote areas for years without their batteries being changed.

In addition to the processing times, we also have to consider the transmission time for the delivery of the message to the server, because the information bit rate is of a few hundred bits per second, and also packet retransmissions take time in case of unsuccessful packet delivery (retransmissions are triggered at specific intervals, for example 10, 30, 60 s). However, the tree-cutting detection scenario has no stringent time constraints, so the achieved alerting times with a few seconds latency is still an effective solution to allow prompt intervention and preserve the environment.

### Findings and Limitations

The main findings achieved in this work are summarized below:

- audio classification on ultra-low-power devices such as ARM M4F microcontrollers is not a trivial task because specific optimizations and pre-processing techniques have to be considered and tested to obtain a good trade-off between accuracy and low calculation and memory resource consumption;
- LoRa technology can actually be adopted for pervasive monitoring in forest and rural scenarios, but vegetation and other obstacles (buildings, hills, etc.) introduce a heavy signal degradation, thus reducing the communication range in case of dense woods or NLoS scenarios;
- keeping in mind these key issues, in this work, a pervasive and accurate tree-cutting audio detection system running on ultra-low-power resource-constrained devices has been successfully implemented, providing a proof of concept also for other audio-based monitoring applications in rural areas and forests scenarios.

In this work, the following limitations have to be considered as well:

- the audio recognition test has only been performed based on recorded sounds in the ESC dataset, so an extensive test of the audio recognition system can be envisaged by listening to real sounds emitted by chainsaws, handsaws, fires, etc. However, a sound detected in a real-life scenario undergoes a sampling and quantization process on the IoT device as it happens for the prerecorded sounds present in the dataset;
- the adopted testbed only envisages measurements in small wood areas for a prevalent rural scenario on sunny weather. Extensive simulations and a deeper empirical validation of the LoRa module transmission under different weather conditions (i.e., rain, snow, wind, fog, etc.) and different vegetation scenarios (i.e., tropical jungle, conifer mountain forests, etc.) could provide more accurate knowledge of LoRa coverage in real-life conditions.

## 6. Conclusions

In this paper, a framework for automatic detection of illegal tree-cutting activity in forests has been introduced and tested. It is based on automatic audio classification, obtained through the adoption of convolutional neural networks. The work has been focused on designing and implementing an efficient neural network, able to obtain good classification accuracy with extremely low processing, memory, and energy consumption.

This goal has been achieved by designing, implementing, testing and evaluating different audio pre-processing techniques and a neural network specifically designed for resource-constrained edge devices. Experimental results show that tree-cutting events can be accurately detected through audio classification on IoT devices with limited capabilities, thus offering a considerable reduction in resource consumption as expected by effective and pervasive IoT monitoring systems. Furthermore, the adoption of LoRa communication on edge-computing end nodes enables the possibility of monitoring tree-cutting activities in large areas with long-range communication, as highlighted by results obtained by onsite experimental tests on LoRa propagation.

Given the results achieved in this work, the proposed system represents an interesting demonstration of the feasibility of tree-cutting audio event recognition through edge-computing on very low-power, memory-constrained and long-range IoT battery-powered devices. In future work, such a framework could be extended to other scenarios involving audio and image classification and monitoring systems in several fields of interest, such as smart cities and wildlife preservation.

## Figures and Tables

**Table 1:** Overview on tree-cutting detection contributions in the literature and respective limitations.

Reference	Main Contribution	Main Limitations
[14]	Low-power microcontroller with sound/vibration sensors and Xbee	Effective only with chainsaw sounds, no long-range wireless transmission
[15]	Ultra-low-power device, sound/vibration sensors, Zigbee with fog computing	Effective only with chainsaw sounds, no long-range wireless transmission
[16]	Low-power microcontroller with sound/vibration sensors and GSM communication	Threshold-based approach, no low-power wireless transmission
[17]	Detection and location of chainsaws through air/soil sound TDOA	Effective only with chainsaw sounds, no wireless communication
[7]	Arduino/Raspberry Pi sound detector with LoRa communication	Effective only with chainsaw sounds, medium–low-power hardware (i.e., Raspberry Pi)
[28]	Chainsaw sound detection adopting spectrograms	Effective only with chainsaw sounds, no details are given on electronics and communication
[29]	92% accuracy on axe stroke sound detection through Gaussian mixture model, K-means Clustering, and Principal Component Analysis.	Effective only with axe stroke sounds, no details on electronics and communication
[18]	94.4% accuracy on chainsaw sound detection through neural networks, WiFi and ZigBee communication	Server-side classification and short-range wireless protocols
[30]	94% accuracy on chainsaw through Neural networks, chainsaw location through TDOA	Medium–low-power hardware (i.e., Raspberry Pi), no long-range communication (i.e., 802.15.4)

**Table 2:** Performance comparison adopting different pre-processing techniques.

Method	Processing Time	Peak RAM
Linear Spectrogram	714 ms	208 kB
Mel Spectrogram	1414 ms	114 kB
MFCC	928 ms	46 kB

**Table 3:** CNN performance comparison adopting different pre-processing techniques.

CNN	Inferencing Time	Peak RAM	ROM	Accuracy
Spectrogram-32	14126 ms	406.0 kB	289.8 kB	71.77%
Spectrogram-8	3001 ms	104.6 kB	96.6 kB	54.31%
Mel spectrogram-32	4132 ms	402.9 kB	144.3 kB	65.19%
Mel spectrogram-8	878 ms	103.8 kB	60.2 kB	64.63%
MFCC-32	1089 ms	203.9 kB	93.9 kB	85.37%
MFCC-8	232 ms	54.1 kB	47.6 kB	85.03%

**Table 4:** RSSI average values obtained for LoS locations.

Location	Distance from Gateway	Average RSSI
L_1-LoS	1 km	−104 dBm
L_2-LoS	2 km	−106 dBm
L_3-LoS	3 km	−108 dBm
L_4-LoS	4 km	−113 dBm
L_6-LoS	6 km	−116 dBm
L_8-LoS	8 km	−121 dBm
L_9-LoS	9 km	---

**Table 5:** RSSI average values obtained for partial blockage locations (NLoS1).

Location Code	Distance from Gateway	Average RSSI
N1_0.5-NLoS type1	0.5 km	−102 dBm
N1_1-NLoS type1	1 km	−111 dBm
N1_1.5-NLoS type1	1.5 km	−116 dBm
N1_2-NLoS type1	2 km	−119 dBm
N1_2.5-NLoS type1	2.5 km	−122 dBm
N1_3-NLoS type1	3 km	---

**Table 6:** RSSI average values obtained for full blockage locations (NLoS2).

Location Code	Distance from Gateway	Average RSSI
N2_0.5-NLoS type2	0.5 km	−119dBm
N2_0.75-NLoS type2	0.75 km	−121 dBm
N2_1-NLoS type2	1 km	---
